# An unusual case of melanoma metastasis in the buccal space: learning by mistakes to distinguish it from salivary neoplasms

**DOI:** 10.1007/s11282-020-00470-x

**Published:** 2020-08-09

**Authors:** P. Grillo, A. P. Savoldi, R. Di Meo, G. Granata, G. M. Rodà, G. Arrigoni, A. M. Saibene, G. Franceschelli, F. Patella, M. Cariati

**Affiliations:** 1 School of Radiodiagnostics, University of Milan, Via Festa del Perdono 7, 20122 Milan, Italy; 2 Unit of Pathology, IRCCS San Raffaele Scientific Institute, Milan, Italy; 3 Otorhinolaryngology Unit, San Paolo Hospital, Via A. di Rudinì 8, 20142 Milan, Italy; 4 Diagnostic and Interventional Radiology Service, San Paolo Hospital, Via A. di Rudinì 8, 20142 Milan, Italy

**Keywords:** Melanoma metastasis, MRI features, Melanoma, Buccal fat pad

## Abstract

**Background:**

The buccal space is an unusual location of malignancies. We report here the case of a woman with a melanoma metastasis in buccal fat pad, to evaluate the imaging features which might lead to the correct, although uncommon, diagnosis.

**Case presentation:**

A 71-year-old woman presented with a painless visible swelling of the left cheek. MRI revealed the presence of a solid lesion located in the buccal fat pad with features suggestive of malignancy. It showed T1 hyperintensity and T2 hypointensity, and restriction of diffusion. Histological examination showed neoplastic cells compatible with melanoma.

**Discussion:**

The lesion features (T1 hyperintensity and T2 hypointensity) initially lead our team to believe that there was a hemorrhagic component, possibly a residue of the biopsy. However, when associated with other malignancy features, such as low apparent diffusion coefficient (ADC) values and contrast enhancement, they should evoke the suspect of melanoma, provided that no biopsy was performed and no trauma occurred in the 3–7 days before.

## Introduction

**Fig. 1:**
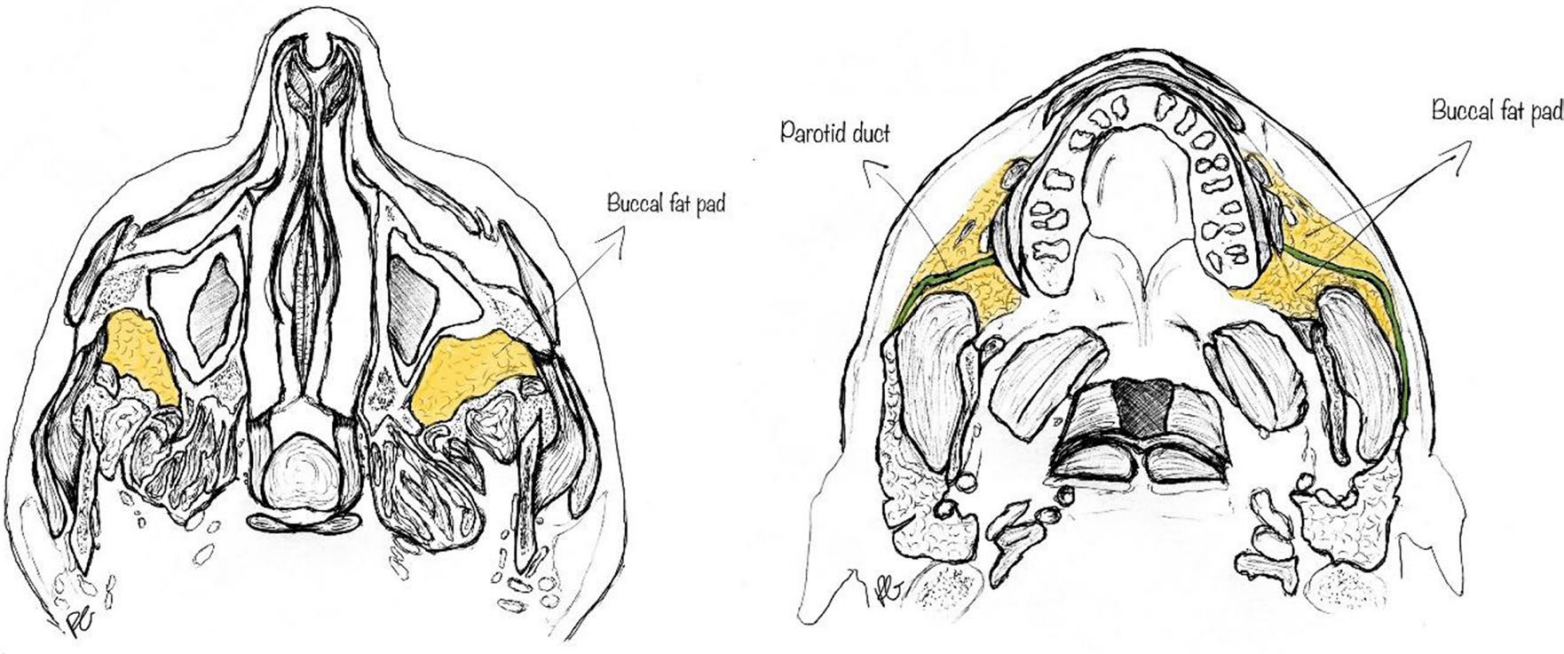
Anatomy of the buccal space

The buccal space is one of the deep anatomical compartments of the head and neck regions, enveloped by the superficial layer of the deep cervical fascia (Fig. 1). It is delimited medially by the buccinator muscle, laterally by the platysma muscle, anteriorly by the mimic muscles, and posteriorly by the mandible, medial and lateral pterygoid muscles, and parotid and masseter muscle [1, 2].

Most of the buccal space is occupied by adipose tissue—the buccal fat pad—which laterally follows the parotid duct ending near the anterolateral portion of the parotid gland; medially, it projects towards the mandible and maxillary sinus; superiorly, it is divided by the temporal muscle into a superficial and deep portion and by the parotid duct into an anterior and posterior portion [3].

The buccal space frequently communicates with the posterior masticatory space and with the infratemporal space; moreover, there is no real lower boundary with the submandibular space. This anatomical space can thus function as a conduit to spread infectious or tumoral tissue between the mouth and the parotid gland.

Buccal space lesions are usually associated with cheek mass or facial swelling [4].

Beside inflammatory manifestations and developmental lesions such as accessory parotid tissue (present in up to 20% of the population), dermoid cysts, and vascular malformations, a variety of tumors can occur in the buccal space [5].

The most common tumor processes localized in this area arise from minor salivary glands such as pleomorphic adenoma (PA), adenoid cystic carcinoma, acinic cell carcinoma, and mucoepidermoid carcinoma. Other tumors can originate from connective, muscular, neural, and lymphatic tissue, such as rhabdomyosarcomas and neurofibromas [5].

Metastatic lesions in this region are a very rare occurrence, accounting for less than 1% of oral cavity malignancies [6]. They are most commonly located in bony structures, particularly the mandible, while only about 33% are found in soft tissues [7]. Metastatic lesions can often be the first sign of a widespread malignant disease, generally indicating poor outcome [8]. However, there are only a few studies focusing on metastatic lesions in the buccal and masticatory space, and even less literature regarding malignant melanoma metastases in this region; moreover, most studies were carried out from a surgical standpoint [9–13].

We report here the case of a female patient, with negative oncological history, who presented to our unit with a swelling of the left cheek, which revealed to be a melanoma metastasis only after surgical exeresis.

We analyze our experience with a revision of the literature, aiming for a retrospective evaluation of the diagnostic imaging characteristics which might be useful in guiding the differential diagnosis, to set an example to avoid repeating the same error in the future and to be of help to those who will find themselves in such a situation.

## Case report

A 71-year-old woman presented with a painless visible swelling of the left cheek. Skin and oral mucosa were intact and of normal color. At palpation, the mass was mobile and apparently not adherent to the cutaneous and mucosal tissue.

For what concerns comorbidities, the patient was only known for Hashimoto thyroiditis in hormone replacement treatment (no relevant comorbidity was known).

Under ultrasound guidance, a 3-cm hypoechoic lesion was visualized in the left malar region and fine-needle-aspiration biopsy (FNAB) was performed. Cytological examination showed numerous Red Blood Cells, some lymphoid small B- and T-cells, plasma cells with no polyclonal atypia and a population of small-sized cells with plasmacytoid cells with the following immunophenotype: S-100 protein + , GFAP ± , P63 ± , cytokeratin7-, suggesting a salivary origin. However, a more precise diagnosis was not possible because of the small amount of material available.

**Fig. 2:**
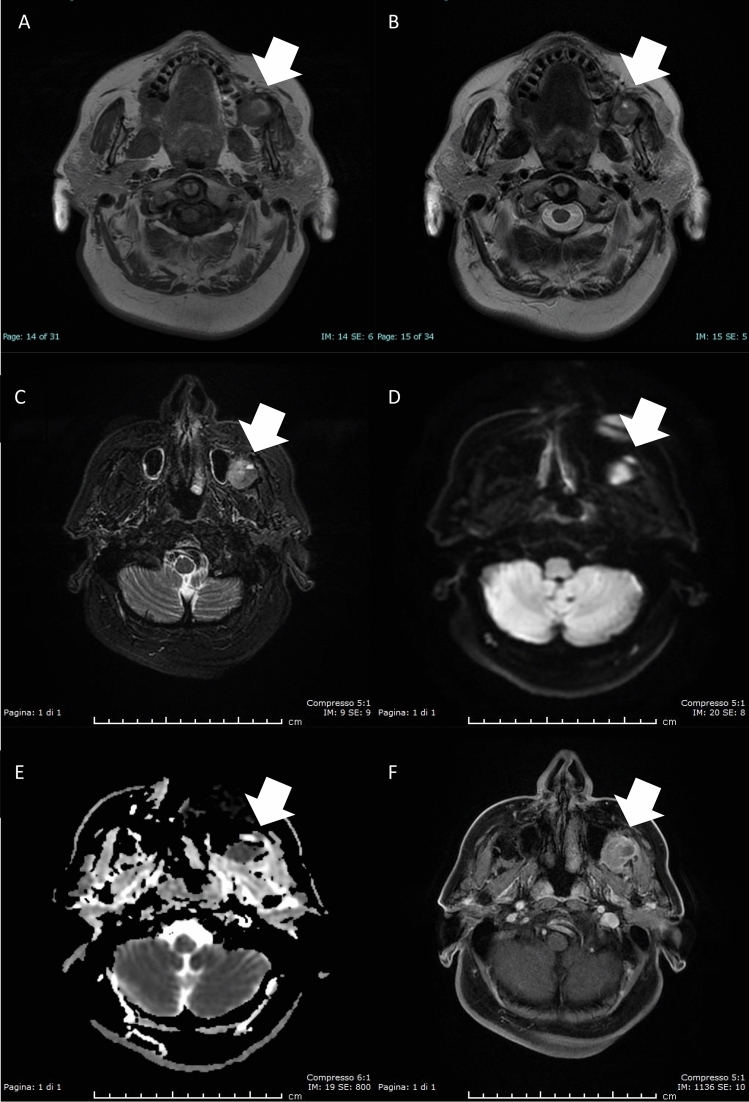
Magnetic resonance imaging revealed a solid mass located in the left buccal space. **a** The lesion (arrow) showed a focal component with high signal intensity on axial T1-weighted imaging and **b** low signal intensity on T2-weighted imaging and **c** on fat-saturated T2 STIR sequences. **d** Diffusion-weighted imaging revealed a restricted diffusion change with **e** low apparent diffusion coefficient of 0.9. **f** After intravenous contrast administration, the lesion showed increased signal intensity on T1-weighted imaging

The following month, Magnetic Resonance Imaging (MRI) with intravenous paramagnetic contrast (Gadobutrol) was performed (Fig. 1). T1-weighted (T1w), T2-weighted (T2w), fat-saturated Short-TI Inversion Recovery (STIR), Diffusion-Weighted Imaging (DWI), and T1w sequences after contrast administration were acquired. It revealed the presence of a solid polylobate lesion located around the left parotid duct, with an antero-inferior component of 13 × 13 × 15 mm in the soft tissues adjacent to the buccinators, and another component of about 22 × 27 × 29 mm in the buccal fat pad (Fig. 2) extending into the infratemporal fossa up to the pterygo-maxillary fissure, which exerted a mass effect on the masticatory muscles and the posterolateral wall of the maxillary sinus. The mass showed contrast medium uptake and diffusion restriction (apparent diffusion coefficient, ADC = 0.9). Notably, the lesion displayed hyperintense signal compared to muscles in T1w sequences and hypointensity on T2w sequences. Some enlarged oval lymph nodes with progressive contrast enhancement were present in the left fifth cervical level. Overall, the MRI findings were suggestive of a malignant lesion that the radiologists assumed to have arisen from minor salivary glands on the basis of the anamnesis of the patient and of the epidemiology of neoplasms in this region.

The T1 hyperintensity was referred to possible blood residues in consideration of the recent bioptic procedure.

Surgical exeresis was performed 2 weeks later. Intraoral access through the gingival fornix was obtained. The lesion, which extended to the pterygo-maxillary fissure, did not adhere to the surrounding tissues and was easily removed. It was partially solid, with a colliquated component.

**Fig. 3:**
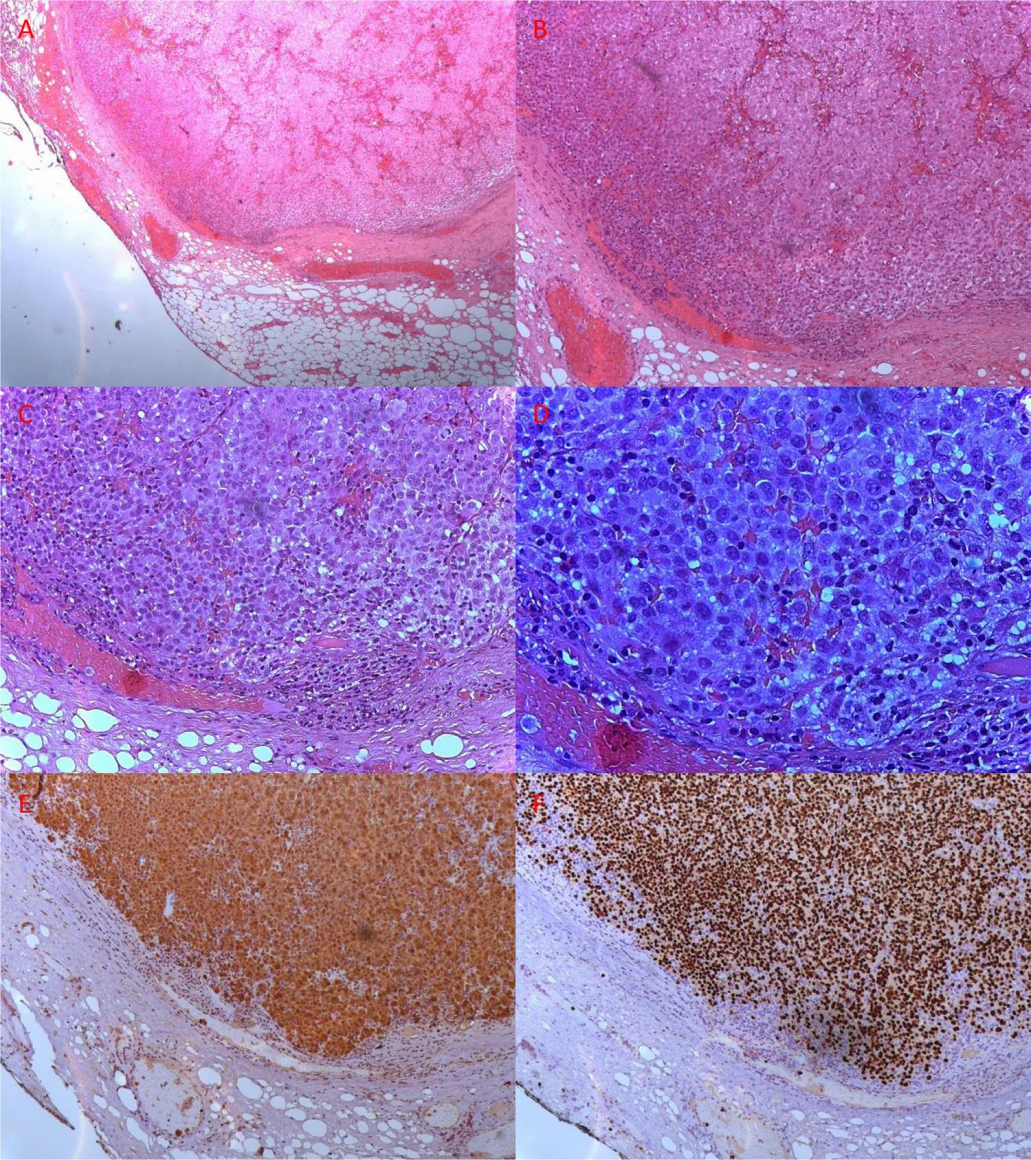
Microscopic pictures of the surgical specimen. **a**–**d** H&E stain at increasing magnification (respectively, × 40, × 100, × 200, and × 400) showing proliferating atypical cells containing coarse-brownish granules suspected to be melanin; **e**, **f**, respectively, S-100 and SOX10 immunohistochemical stains confirming the diagnosis of melanoma

Histological examination showed the presence of fibro-connective and lymphatic tissue containing malignant neoplastic cells compatible with a nodule of melanoma inside the buccal fat pad (Fig. 3).

Gene mutation analysis for c-KIT and BRAF was performed: c-KIT was Wild Type (WT), while V600 mutation of BRAF exon 15 was found.

**Fig. 4:**
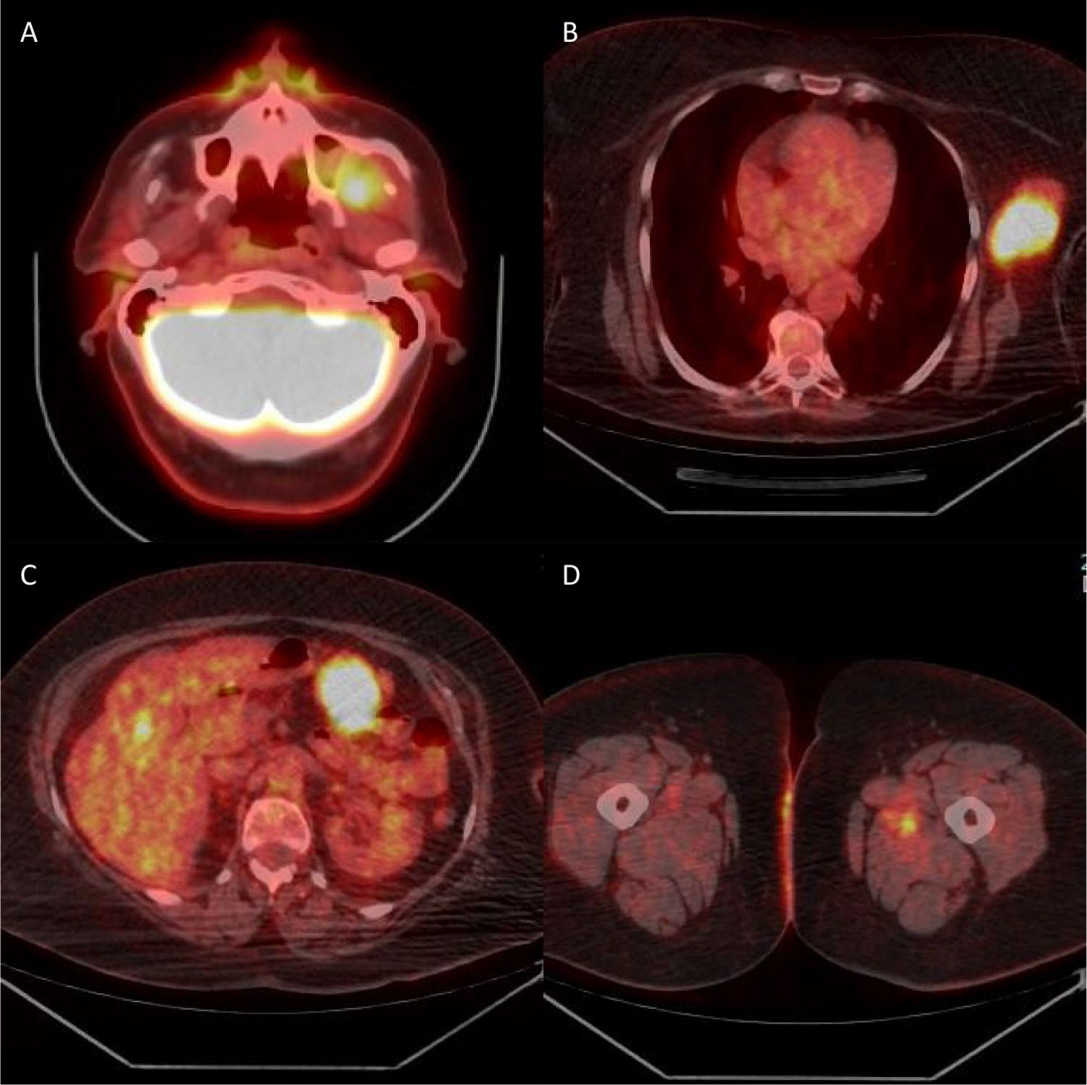
[F-18]FDG PET-CT reveals avid FDG uptake by the left buccal space lesion (**a**). Multiple pathological radiotracer hyper-accumulation localizations were present, such as in a voluminous left axillary lymph node (**b**), a perigastric solid lesion (**c**), and in the muscular tissue of the left thigh (**d**)

[F-18]FDG PET-CT (Fig. 4) revealed multiple localizations of pathological radiotracer hyper-accumulation: bilateral suprahyoid and axillary lymph nodes, fifth liver segment, right adrenal gland, multiple abdominal solid lesions (perigastric, left psoas muscle, right iliac fossa), various subcutaneous, and muscular tissue localizations (left and right thigh and gluteus, right pectoral muscle, right masseter, and right fourth toe).

Full-body computed tomography (CT) with intravenous contrast was then performed, with the following results: no encephalic lesions; 2-cm left cervical lymph node (LN); 5-mm left paracardiac micronodule; 7-cm LN conglomerate in the left axillary region; bilateral adrenal lesions (3.5 cm on the right side; 2 cm on the left); multiple enlarged mesenteric LNs, the largest of about 2 cm; 2.8-cm lesion in the left psoas muscle.

Dermatological examination revealed a 2-cm ulcerated cutaneous lesion on the distal phalanx of the left fifth finger, compatible with a primitive localization. The patient reported having it for about 1 year but never investigating it. Incisional biopsy of the lesion was performed, and histological examination confirmed the presence of melanoma cells with V600E mutation of BRAF gene.

Immediately afterwards, treatment was begun with Dabrafenib 300 mg/die and Trametinib 2 mg/die.

Two follow-up full-body CTs at 2 and 6 months were performed and showed significant size reduction of the axillary and abdominal LNs, of the adrenal and mesenteric localizations, and disappearance of the thoracic nodules.

## Discussion

A variety of processes can occur in the buccal space, such as infections, developmental lesions, and tumoral processes.

Neoplastic lesions in this area origin most commonly from minor salivary glands, such as pleomorphic adenoma, adenoid cystic carcinoma, acinic cell carcinoma, and mucoepidermoid carcinoma.

The most frequent benign glandular tumor is PA, which consists of both mesodermal and glandular tissue [3]. It has smooth, rounded margins and shows low signal intensity on T1w image, high T2 signal intensity, and no diffusion restriction on DWI.

The most common malignant glandular tumor is adenoid cystic carcinoma. Some authors have suggested that lesions with low or intermediate T2-hyperintensity or invading surrounding tissues tend to be more aggressive, whereas lesions characterized by high signal intensity in T2w sequences have lower cellularity and better prognosis [14].

Other tumors can originate from connective, muscular, neural, and lymphatic tissue, such as rhabdomyosarcomas and neurofibromas [5]; rhabdomyosarcomas appear as muscle density masses at CT and are hyperintense relative to muscles on T2w MRI often showing bone destruction [15], while neurofibromas show low intensity on T1w imaging and high intensity on T2w imaging and are often associated with neurofibromatosis. Single neurofibromas characteristically show the target sign with peripheral hyperintensity [15].

Usually, in diffusion-weighted MRI, the ADC of malignant neoplasms of the head and neck regions is lower than that of benign tumors, and it is an important tool in guiding the diagnostic process [16].

Metastatic malignant melanoma is an uncommon head and neck neoplasm. The most frequent localizations of melanoma metastases are lymph nodes (73.6%), lungs (71.3%), liver (58.3%), brain (49.1%), bone (48.6%), heart (47.2%), adrenal glands (46.8%), and gastrointestinal tract (43.5%) [17].

Melanoma localized in the oral cavity and surrounding anatomical regions, such as the buccal fat pad, accounts for 0.2–8% of the total cases of melanomas of the body [18]. However, most studies focus on primary malignant melanoma, while secondary localization of melanoma in this region has been very rarely reported in the literature, with only one study describing a metastatic melanoma in the tongue and, to the best of our knowledge, no reports at all of the secondary melanomas in the buccal space [19].

Therefore, based on epidemiologic criteria, it is rather hard to diagnose metastatic melanoma in the buccal space in patients with an unknown primary tumor, as in our case.

Yet, MRI may be helpful in the identification of melanoma metastases [20].

Two main MRI patterns of melanoma can be identified: a melanocytic and amelanocytic pattern [21].

Lesions showing a melanocytic pattern, which is the more common one, are characterized by signal hyperintensity in T1w and hypointensity in T2w images. This is due to the presence of melanin and blood products inside the lesion, although some authors argue that the T1 hyperintensity is more relevantly associated with the presence of blood products [21–23].

Less commonly, malignant melanoma may show an amelanocytic (and therefore a specific) pattern, characterized by hypo- or isointensity in T1w images and hyper- or isointensity in T2w images [21], which is associated at histopathological examination to lower quantity of melanin inside the lesion [24].

Macroscopically, metastatic malignant melanoma may present in different ways, ranging from small rapidly growing lesions which may initially go undetected or misrecognized as artifacts, to showing a military pattern [25].

In our case, MRI showed a single lesion that exhibited diffusion restriction with ADC of 0.9 and high contrast medium uptake, characteristics suggestive of malignancy as it was proposed in the report.

Moreover, the mass was characterized by T1 hyperintensity and T2 hypointensity, a behavior which may be attributed to the melanoma melanocytic pattern; however, it is not specific, since such features may be ascribed to other paraphysiological and pathological processes, including hemorrhages and proteinic fluid collections.

In this case, radiologists supposed that there was a hemorrhagic component inside the lesion, possibly a residue of the FNAB which had been performed 1 month and a half before. Yet, this was a mistake. In fact, only early subacute hemorrhage dating 3–7 days is featured by T1 hyperintensity and T2 hypointensity, whereas chronic hemorrhages dating more than 1 month are rather characterized by both T1 and T2 hypointensity (26).

Overlooking this aspect and ignoring a part of the anamnesis, radiologists assumed that they were dealing with a primitive buccal space neoplasm, namely with a malignant (minor) salivary gland tumor.

On the contrary, this case teaches that T1 hyperintensity and T2 hypointensity, when associated with other malignancy features in buccal space lesion such as low ADC values and contrast enhancement, should always evoke the suspicion of melanoma, provided that no biopsy was performed and no trauma occurred in the 3–7 days before MRI examination.

## Abbreviations

ADC: Apparent diffusion coefficient
CT: Computed tomography
DWI: Diffusion-weighted imaging
FNAB: Fine-needle-aspiration biopsy
LN: Lymph node
MRI: Magnetic resonance imaging
PA: Pleomorphic adenoma
STIR: Short-TI inversion recovery
T1w: T1-weighted
T2w: T2-weighted
